# Box-Behnken Design Assisted Optimization and Characterization of Chitosan Film for Simultaneous Topical Delivery of Ascorbic Acid and Metronidazole

**DOI:** 10.3390/pharmaceutics17050562

**Published:** 2025-04-24

**Authors:** Bilawal Khan, Pakorn Kraisit, Supaporn Santhan, Namon Hirun

**Affiliations:** 1 Division of Pharmaceutical Sciences, Faculty of Pharmacy, Thammasat University, Pathumthani 12120, Thailand; bilawal.kha@dome.tu.ac.th (B.K.); pakorn54@tu.ac.th (P.K.); 2 Thammasat University Research Unit in Smart Materials and Innovative Technology for Pharmaceutical Applications (SMIT-Pharm), Faculty of Pharmacy, Thammasat University, Pathumthani 12120, Thailand; kdoublee.kee@gmail.com

**Keywords:** chitosan, ascorbic acid, metronidazole, topical film

## Abstract

**Background/Objectives:** The objective of this study was to develop chitosan films plasticized with glycerol for the topical delivery of ascorbic acid and metronidazole. **Methods:** The films were prepared using a casting technique in which an aqueous ascorbic acid solution served as the solvent, eliminating the need for additional mineral or organic acids. The influence of compositions on film characteristics—specifically mechanical properties and surface pH—was examined, and an optimized formulation was identified using a Box-Behnken design-response surface methodology. Relevant characterization techniques and in vitro evaluations were conducted to assess the properties and performance of the optimized film formulation. **Results:** Results showed that both glycerol and ascorbic acid contributed to the plasticization of the films. Fourier-transform infrared spectroscopic analysis of the optimized film revealed the formation of chitosan ascorbate and interactions between chitosan and glycerol. In addition, the thermogram and powder X-ray diffractogram demonstrated alterations in the thermal behavior and crystallinity of the embedded bioactive compounds. The developed film possessed the preferred swelling capacity. Moreover, in vitro release studies revealed a co-release pattern, delivering both bioactive compounds simultaneously. **Conclusions:** These findings suggest that the prepared chitosan-based film could serve as a promising platform for topical delivery.

## 1. Introduction

The functions of the skin are vital for human survival. The skin is the primary barrier against environmental stressors, such as harmful chemicals and pathogens [1]. Cutaneous injuries, such as chronic wounds and skin wound infections, impair the skin’s defense against harmful exterior assaults. Patients with infected wounds often experience complications that can diminish the quality of life, elevate the financial burden, and increase the risk of death [1,2]. The drug delivery approaches that provide a protective layer and enable the release of bioactive molecules to the skin lesion represent a promising candidate for cutaneous wound treatment [3]. Topical drug delivery, which is primarily designed to be applied externally to the body and to deliver therapeutic agents to specific target sites for localized effects [4], offers several notable advantages over oral and parenteral routes of administration, including enabling high drug levels at the skin target site, avoiding systemic exposure, minimizing off-target adverse effects, and reducing dosage requirements for effective cutaneous delivery [5]. Developing various topical materials for wound treatment represents a significant challenge in the medical and pharmaceutical fields. The fabrication of polymeric films is a promising strategy for topical drug delivery to combat local diseases and impairments [6,7]. Films designed with mechanical properties in the appropriate range and consistent with the skin’s strength and flexibility are preferable, as they can adapt to the physical features of application sites and facilitate the application on dermal surfaces [8]. Among the diverse polymers employed as film formers, polysaccharides stand out as promising biopolymers for the fabrication of topical films in pharmaceutical and biomedical contexts, owing to their biocompatible and biodegradable nature, as well as their ease of processing and moldability [2]. Changes in composition and the potential interactions among components can influence the film’s properties. Understanding the influence of compositions and their content on the relevant characteristics of the film is crucial and can facilitate the fabrication of the polysaccharide-based film for topical application.

**Figure 1:**
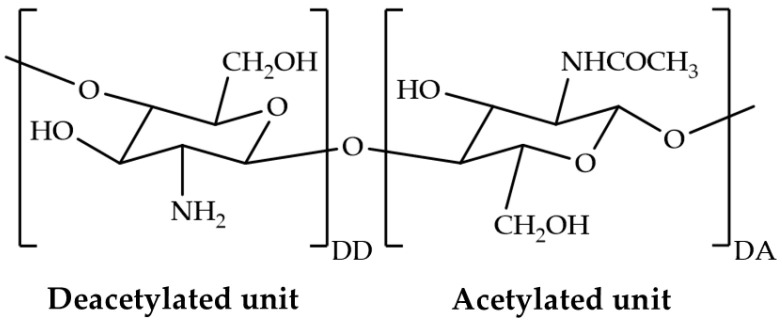
Chemical structure of chitosan, defined by its degree of deacetylation (DD, %) and degree of acetylation (DA, %).

Chitosan is a linear polysaccharide derived from the partial deacetylation of chitin [3]. This polymer consists of D-glucosamine and N-acetyl-D-glucosamine units linked by β-(1–4) glycosidic bonds [9]. Chitosan is predominantly composed of monomer units with the general formula C_6_H_11_O_4_N, which are derived from the deacetylation of repeating units in chitin [10,11]. The chemical structure of chitosan is illustrated in Figure 1. Based on molecular weight, chitosan can be classified into four distinct types: low molecular weight (<190 kDa), medium molecular weight (190–500 kDa), high molecular weight (500–1000 kDa), and ultrahigh molecular weight (>1000 kDa) [11]. Chitosan possesses film-forming ability and has attracted much attention for topical applications due to its biocompatibility, biodegradability, non-toxicity, and biological activities, such as antimicrobial activity and potential wound healing activity [6]. Chitosan is distinguished from most other natural polysaccharides by its primary amine groups, which confer distinctive properties. However, chitosan exhibits limited solubility in water and most organic solvents due to its inter- and intramolecular hydrogen bonding and semicrystalline structure, thereby restricting some potential applications [12]. This polymer preferentially dissolves in certain dilute acidic solutions. In acidic environments, the protonation of its amino groups causes electrostatic repulsion between the positively charged polysaccharide structure, allowing chitosan to dissolve. Owing to the presence of positively charged residues and functional groups capable of donating and accepting hydrogen bonds, chitosan can interact with a wide variety of polymers, both ionic and nonionic, as well as some small molecules [6,13]. To date, acetic acid is the most often utilized solvent for preparing aqueous solutions of chitosan. However, the films cast from chitosan solutions in acetic acid displayed the characteristics of brittle materials [14]. Although chitosan films possess several advantageous properties, they tend to be rigid in their unmodified form and, therefore, require plasticizers to tailor their physico-mechanical characteristics [15]. It is possible to modify the properties of chitosan films by altering the type of acid as distinct interaction patterns emerge between chitosan and acid molecules. Nevertheless, there is limited information on how interactions with organic acids other than acetic acid influence the structure and characteristics of chitosan films [14].

**Figure 2:**
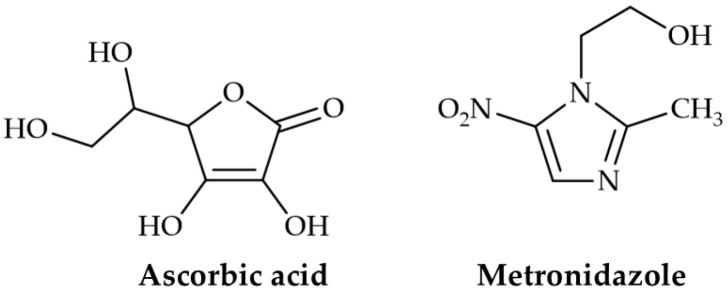
Chemical structures of ascorbic acid and metronidazole.

Ascorbic acid, a sugar acid, is an essential dietary nutrient involved in biochemical and physiological processes. The molecular formula and molecular weight of ascorbic acid are C_6_H_8_O_6_ and 176.1 g/mol, respectively. Its chemical structure is shown in Figure 2. It has a beneficial effect on the formation of collagen, blood vessels, and skin, as well as wound repair [16]. Besides the benefit from oral administration, topical application of the drug delivery matrix loaded with ascorbic acid confers the potential action for tissue healing [2]. In addition to its remarkable bioactivity, ascorbic acid is also considered a potential organic acid to promote the dissolution of chitosan in an aqueous solution. An ascorbic acid-containing solution in water has been explored for its ability to form a complex with chitosan to fabricate cosmetic materials and edible films for food packaging [17,18]. Therefore, ascorbic acid should be a viable candidate for use as a component of the acidic medium for the further development of drug-loaded chitosan films for topical delivery.

Metronidazole, a nitroimidazole derivative, is recognized as the potential drug of choice for the treatment of skin and soft tissue infections [19]. The molecular formula and molecular weight of metronidazole are C_6_H_9_N_3_O_3_ and 171.2 g/mol, respectively. Its chemical structure is depicted in Figure 2. The local effect of metronidazole on the skin lesion is promising, as it controls the odor of the infected wound and has a favorable influence on skin healing [20]. In this regard, the topical delivery of metronidazole would favor drug release at the target wound site.

The biological activities of both small molecules, ascorbic acid and metronidazole, make the development of chitosan film containing these bioactive molecules particularly intriguing for fabricating a co-delivery system. Because ascorbic acid itself has biological activity and can be used for film preparation without the need for other mineral acids or organic acids, it is eco-friendly to make chitosan film for the delivery and release of both small molecules to the skin wound site. This green approach is devoid of the multiple types of compounds used for solubilizing the film-forming excipient. Glycerol, a non-toxic and eco-friendly polyol derived from the oleochemical production process [21,22], may also be used as a plasticizer to tailor the physico-mechanical characteristics of the topical film.

Multiple composition factors may influence the film features, and the response surface methodology (RSM) for experiment design provides a viable approach for concurrently assessing variables when complex interactions arise [23,24]. The relationship between experimental responses and factors in complex systems can be assessed using a Box-Behnken design–response surface methodology (BBD-RSM) [24,25]. This method can reduce the number of experimental sets without compromising accuracy compared to traditional factorial design methods [25,26]. Consequently, the application of the BBD-RSM enables the investigation of critical material constituents and their interplay, facilitating the development of the film.

This study presents a promising approach for preparing topical film by incorporating ascorbic acid and metronidazole into chitosan film without adding other mineral acids or organic acids. The films comprising chitosan, ascorbic acid, metronidazole, and glycerol, the non-toxic and eco-friendly plasticizer, were fabricated, and their physico-mechanical features were characterized. In order to comprehend the influence of constituents on the film characteristics, BBD-RSM was utilized. Then, the optimized preparation was selected. The thermal behavior, crystallinity, and potential molecular interactions within the film compositions were analyzed using differential scanning calorimetry, X-ray diffractometry, and Fourier transform infrared spectroscopy, respectively. The optimized film was also examined for its swelling and release characteristics.

## 2. Materials and Methods

### 2.1. Materials

Low molecular weight chitosan with a deacetylation degree of 90% and ascorbic acid were supplied by Sisco Research Laboratories Pvt. Ltd. (Mumbai, India). Metronidazole and glycerol were obtained from PC Drug Center Co., Ltd. (Bangkok, Thailand). Bovine serum albumin (BSA) was purchased from Fisher BioReagents (Thermo Fisher Scientific, Waltham, MA, USA). HPLC-grade methanol was obtained from Macron Fine Chemicals (Avantor Performance Materials, Radnor, PA, USA). Cellulose membranes (SnakeSkin^TM^ dialysis membrane, MWCO = 10 kDa) were obtained from Thermo Fisher Scientific (Waltham, MA, USA). Water utilized in this study was purified through a Milli-Q^®^ Advantage A10 Water Purification System (Merck KGaA, Darmstadt, Germany). All other chemicals used were of analytical grade.

### 2.2. Response Surface Methodology (RSM)

The experimental design and data analysis were performed using Design-Expert^®^ software (version 13; Stat-Ease, Inc., Minneapolis, MN, USA). In this study, the Box-Behnken design-response surface methodology (BBD-RSM) was used to assess the influences of chitosan (X_1_), ascorbic acid (X_2_), and glycerol (X_3_) on the mechanical properties, including ultimate tensile strength (Y_1_) and elongation at break (Y_2_), as well as pH (Y_3_) of prepared films. X_1_ and X_2_ were expressed in % *w*/*w* relative to the total weight of the film-casting solution, while X_3_ was expressed in wt% based on chitosan content. To ensure suitability for film formation, the concentrations of the polymer and the studied components should fall within a range that prevents the film-casting solution from becoming excessively fluid or overly viscous [27]. Based on our preliminary study, in which the flowability of the solution was visually assessed by tilting the container, the appropriate concentration or quantity ranges for each investigated factor in the film-casting solution were defined. As presented in Table 1, the independent factors were examined at three levels: high (+1), medium (0), and low (−1). As shown in Table 2, 17 experimental formulations—including five center points—were generated. Each experimental response was measured in triplicate for each run, and then the mean values were used to model the relationship between the independent factors and the responses. Analysis of variance (ANOVA) was conducted to determine the model’s statistical significance. The model’s suitability was further assessed using the *p*-value (*p* < 0.05), the coefficient of determination (R^2^), the adjusted coefficient of determination (adjusted R^2^), and the predicted coefficient of determination (predicted R^2^). In addition, the appropriate model was required to exhibit a non-significant lack of fit.

### 2.3. Film Preparation

The film samples were prepared using a film-casting method. The contents of the three constituents in the film-casting solution were varied according to the experimental design described in the previous subsection. To prepare the polymer solution, the required amount of ascorbic acid was dissolved in water under continuous stirring, followed by gradually adding the necessary amount of chitosan. The resulting dispersion was stirred for 3 h to ensure complete dissolution. Metronidazole was added to achieve a concentration of 0.5% *w*/*w* relative to the total film-casting solution weight. Next, the necessary quantity of glycerol was introduced, and stirring was continued to ensure homogeneity. Subsequently, 15 g of each prepared film-casting solution was poured into a Petri dish. The samples were dried in an oven at 35 °C for 18 h, and then the obtained films were removed and stored for further characterization.

### 2.4. Mechanical Test

A TA-XT plus texture analyzer (Stable Micro Systems, Godalming, UK) was used to evaluate the mechanical properties, including ultimate tensile strength and elongation at break. The films were cut into rectangular pieces with 1 cm × 5 cm dimensions. Then, the 1 × 1 cm^2^ sections at each end were secured with clamps. Therefore, the effective testing area was 1 × 3 cm^2^. The films were stretched in tension mode at a rate of 0.5 mm s^−1^ [28]. All experiments were conducted in triplicate.

### 2.5. Surface pH Measurement

The surface pH was determined according to the previously described methodology [29]. To allow for swelling, each film sample with an area of 1 × 1 cm^2^ was submerged in a glass tube filled with 5 mL of water. A combined pH electrode was then placed and positioned against the surface of the film to measure pH. The experiment was conducted with three replicates.

### 2.6. Viscosity Measurement

The viscosity of the film-casting solution used to prepare the optimized film was measured in triplicate using a rheometer (HAAKE MARS 40, Thermo Fisher Scientific, Dreieich, Germany) equipped with a cone-plate system (C60 1°/Ti). A shear rate sweep was conducted over the range of 0.1–100 s^−1^ at 25 °C, and the viscosity at a low shear rate of 10 s^−1^ was recorded to assess the suitability of the solution for film casting applications [30].

### 2.7. Fourier-Transform Infrared (FTIR) Spectroscopy

FTIR spectra were acquired using a Bruker Alpha II FTIR spectrometer (Bruker, Bremen, Germany) equipped with a single reflection diamond attenuated total reflection (ATR) accessory. All spectra were collected in the ATR mode by averaging 32 scans at a resolution of 2 cm^−1^.

### 2.8. Differential Scanning Calorimetry (DSC)

DSC thermograms were obtained using a Mettler Toledo DSC 3+ differential scanning calorimeter (Mettler-Toledo, Viroflay, France) under a nitrogen flow of 50 mL min^−1^. Each sample was loaded into the aluminum crucible and subjected to heating with a scanning rate of 10 °C min^−1^.

### 2.9. Powder X-Ray Diffraction (PXRD) Analysis

PXRD patterns were measured using a Rigaku MiniFlex II diffractometer (Rigaku Corporation, Tokyo, Japan) with CuKα radiation (λ = 1.5406 Å) as an X-ray source generated at 40 kV and 15 mA. The samples were scanned in the 2θ range of 3–50° with an increment step of 0.1°.

### 2.10. Swelling Study

The swelling capacity of the film samples was assessed by determining the weight change of the film following immersion in the medium over varying time points [31,32]. The film samples, in a 1 cm × 1 cm shape, were excised from the optimized film formulation and subsequently weighed prior to immersion in 5 mL of simulated wound fluid (SWF), which consisted of 2% *w*/*v* BSA, 0.02 mol L^−1^ CaCl_2_, and 0.4 mol L^−1^ NaCl [31]. The swollen samples were removed from the SWF at predetermined intervals. Excess medium on the surface of the swollen films was carefully blotted using filter paper, and each sample was subsequently weighed. The swelling capacity was determined at 0.5, 1, 1.5, 2, 3, 4, and 6 h. All swelling tests were carried out in triplicate. The swelling capacity, determined by the swelling degree in percentage, can be calculated using the following equation [31,32,33].

(1) $$Swelling\;degree(\%)=\frac{W_{s}-W_{d}}{W_{d}}\times 100$$

where *W_d_* and *W_s_* are the initial weight of the dry film and the weight of the swollen film, respectively.

### 2.11. Drug Content Study

The content of each active compound in the optimized film was determined in triplicate, and the actual drug content was expressed as a percentage of the theoretical content, as previously described [24,34,35]. Briefly, film samples were cut into 1.0 cm × 1.0 cm sections and placed individually into 100 mL volumetric flasks containing a solvent mixture of methanol and phosphate buffer (pH 6.2) in a 70:30 (*v*/*v*) ratio. The samples were sonicated in an ultrasonic bath for 1 h to facilitate drug extraction. The resulting solutions were filtered through 0.22 μm syringe filters and analyzed using high-performance liquid chromatography (HPLC). HPLC analyses were conducted using a Shimadzu UFLC system (Shimadzu, Kyoto, Japan) equipped with a Hypersil BDS C18 column (250 × 4.6 mm id; particle size 5 µm), and the analytes were detected using a diode array detector at 254 nm. A mixture of methanol and phosphate buffer pH 6.2 (70:30 *v*/*v*) was used as the mobile phase with a flow rate of 1 mL min^−1^. The quantities of ascorbic acid and metronidazole were determined by comparing their peak areas with those obtained from standard calibration curves prepared for each compound. The drug content of each active compound was expressed as a percentage, calculated as (actual drug content/theoretical drug content) × 100.

### 2.12. In Vitro Release Study

The in vitro release characteristics of ascorbic acid and metronidazole were assessed using a modified Franz cell approach designed to simulate topical release [36,37]. The receptor compartment was filled with 15 mL of the receptor medium. The receptor medium was SWF, prepared without BSA to avoid obstruction of the HPLC column used in the quantitative analysis by the HPLC assay [38]. The cellulose membrane was pre-equilibrated in the medium before being positioned between the donor and receptor compartments. The circular film sample with a radius of 0.75 cm was then mounted on the cellulose membrane. The temperature of the receptor compartment was controlled at 37 °C using a circulating water jacket. The receptor medium was sampled at predetermined time intervals of 0.5, 1, 1.5, 2, 3, 4, and 6 h. At each sampling time, 0.5 mL of the release medium was collected and replaced with the same volume of fresh medium. The HPLC method was used to separate ascorbic acid and metronidazole in the collected release medium and to perform their quantitative analysis. The HPLC conditions were as described in the preceding subsection. All experiments were performed in triplicate.

## 3. Results and Discussion

### 3.1. RSM Analysis and Optimization

The Box-Behnken design of response surface methodology is widely regarded as a valuable approach for establishing the causal relationship between the studied factors and the responses [26]. The BBD-RSM was employed to assess the influence of the factors on the mechanical properties and pH of the prepared films. This design allows for the evaluation of main effects, interaction effects, and quadratic effects [26].

#### 3.1.1. Influence on Ultimate Tensile Strength (Y_1_)

According to Table 3, the statistical outputs from regression analysis revealed that the effect of the component compositions on the ultimate tensile strength followed a response surface two-factor interaction (2FI) model with R^2^ of 0.9377, a sequential *p*-value of 0.0009, and a lack-of-fit *p*-value of 0.0864. The difference between the adjusted R^2^ of 0.9003 and the predicted R^2^ of 0.7292 was less than 0.2, which is desirable and indicates reasonable agreement for the suggested model [39,40].

**Figure 3:**
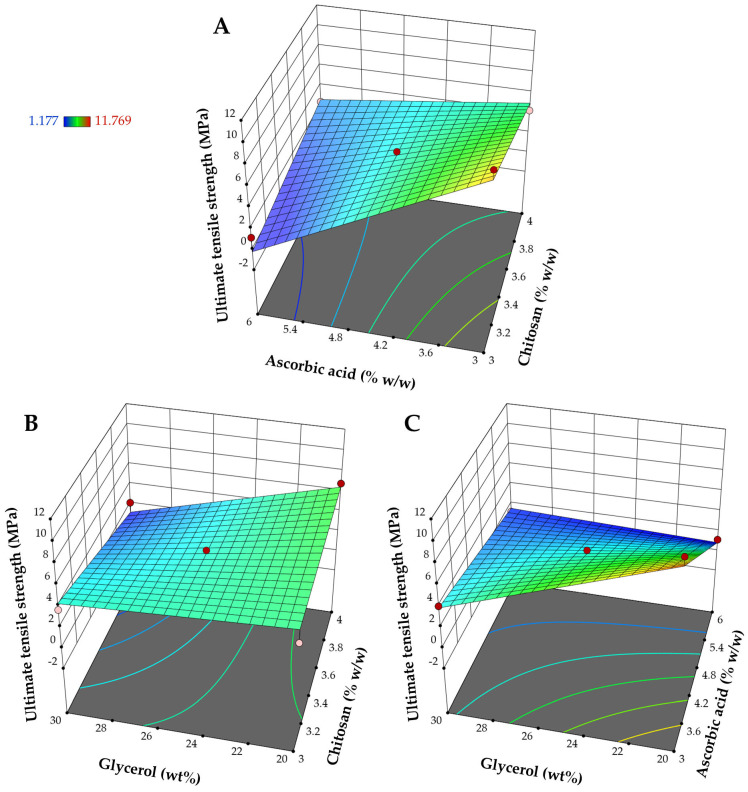
Response surface plots represent the influence of different factors on the ultimate tensile strength: (**A**) impact of chitosan and ascorbic acid at the mid-level of glycerol, (**B**) impact of chitosan and glycerol at the mid-level of ascorbic acid, and (**C**) impact of ascorbic acid and glycerol at the mid-level of chitosan.

Table 4 indicates that all interaction parameters were significant model terms (*p*-value < 0.05). The interaction terms illustrate how the response alters when two factors undergo changes simultaneously [23]. The individual effects of glycerol and ascorbic acid are significant (*p*-value < 0.05), whereas the main effect of chitosan on the ultimate tensile strength is not significant (*p*-value = 0.1487). The effect of these factors on the ultimate tensile strength is depicted in the 3D surface plots shown in Figure 3. Figure 3 and Table 4 demonstrate that ascorbic acid and glycerol have a prominent impact on the ultimate tensile strength. Increasing ascorbic acid tended to cause a significant decrease in the ultimate tensile strength of the films. The observed negative impact of ascorbic acid on the ultimate tensile strength aligns with previous studies, which have shown that the addition of ascorbic acid into biopolymer-based films leads to a reduction in the tensile strength [41,42]. It has been suggested that ascorbic acid can function as a plasticizer, weakening hydrogen bonds between neighboring polymer chains and decreasing the molecular compactness of the polymer network [41,42]. Also, increasing the relative percentage of glycerol to chitosan content significantly decreased the ultimate tensile strength of the obtained films due to the plasticization of the films by glycerol. The formation of hydrogen bonds between glycerol and the polymer chains weakened the direct attraction between the polymer chains [43,44]. As a result, the polymer chains could slip apart more easily during tensile testing, decreasing the tensile strength of the materials. Both ascorbic acid and glycerol are likely to enhance the flexibility of the material due to the plasticization effect, as will be discussed further in the elongation at break results.

#### 3.1.2. Influence on Elongation at Break (Y_2_)

**Figure 4:**
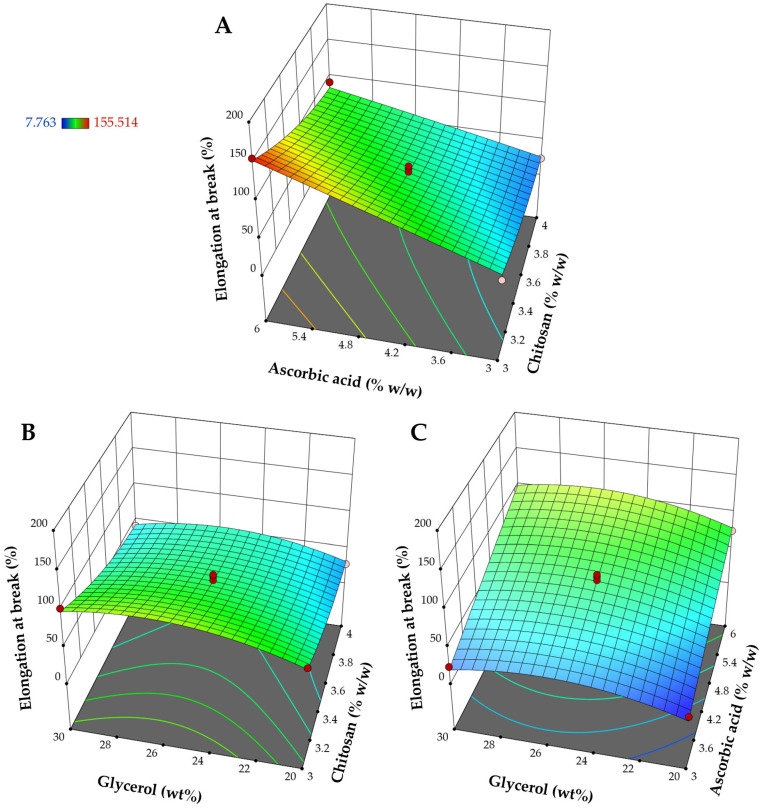
Response surface plots represent the influence of different factors on the elongation at break: (**A**) impact of chitosan and ascorbic acid at the mid-level of glycerol, (**B**) impact of chitosan and glycerol at the mid-level of ascorbic acid, and (**C**) impact of ascorbic acid and glycerol at the mid-level of chitosan.

According to Table 3, the quadratic model, with a R^2^ of 0.9722, a sequential *p*-value of 0.0131, and a lack of fit *p*-value of 0.4495, was the appropriate model for the elongation at break. The adjusted R^2^ of 0.9365 and the predicted R^2^ of 0.7762 are in reasonable agreement. All individual contributions of factors, including X_1_, X_2_, and X_3_, were significant model terms, while only the X_3_^2^ quadratic term was statistically significant, as shown in Table 4. In the analysis of the coefficients of linear parameters regarding coded factors (Table 4) and the influence of the independent variables on the response surface of the elongation at break (Figure 4), chitosan content exhibited a negative effect, whilst the other two independent variables showed a positive impact on the values of elongation at break. Chitosan films are known for their low flexibility and fragility, primarily due to the intermolecular and intramolecular interactions between hydroxyl groups and between hydroxyl and amine groups [45]. The gradual decrease in film flexibility with increasing chitosan concentration may be attributed to the denser intermolecular attractions among polymer chains resulting from the higher chitosan content [46]. The positive impact of ascorbic acid and glycerol on the elongation at break aligns with the previously discussed plasticization effects of these substances. The addition of the plasticizers increased the flexibility of the polymeric films. Consequently, the plasticization rendered the material more ductile, as evidenced by an enhanced deformation capacity and reduced tensile strength [43].

#### 3.1.3. Influence on pH (Y_3_)

**Figure 5:**
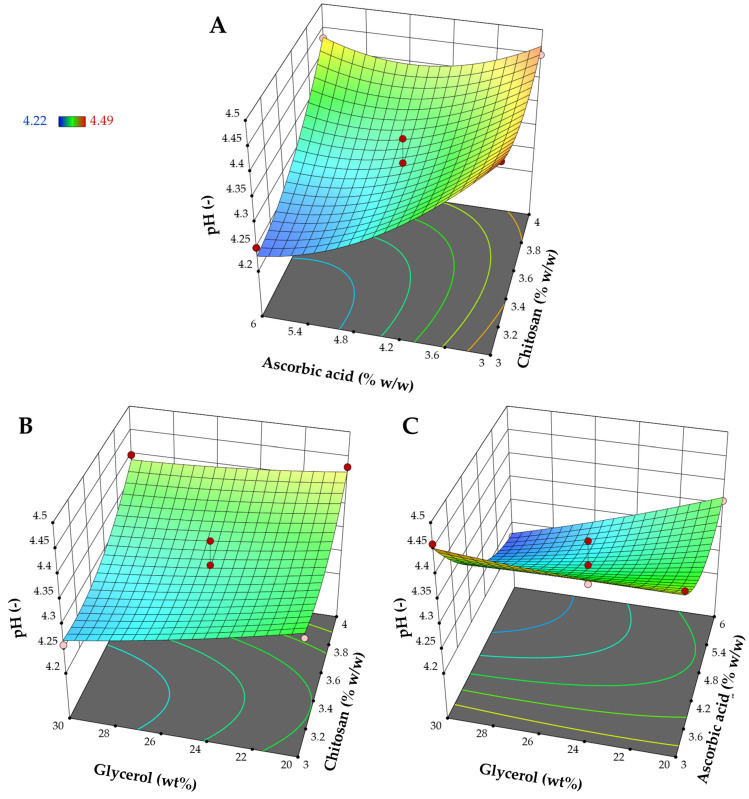
Response surface plots represent the influence of different factors on pH: (**A**) impact of chitosan and ascorbic acid at the mid-level of glycerol, (**B**) impact of chitosan and glycerol at the mid-level of ascorbic acid, and (**C**) impact of ascorbic acid and glycerol at the mid-level of chitosan.

The quadratic model is the most appropriate model for elucidating the impact of the factors on pH values, as indicated in Table 3. Significant model terms included all linear terms (X_1_, X_2_, and X_3_), two interaction terms (X_1_X_2_ and X_2_X_3_), and the quadratic terms of X_1_^2^ and X_2_^2^. The response surface plots (Figure 5) reveal the impact of chitosan, ascorbic acid, and glycerol on pH. Considering the main effect of each component, increasing the amount of chitosan increased pH while increasing the amount of ascorbic acid and the relative amount of glycerol to the total polymer content reduced pH. The amount of chitosan had an impact on the pH of the chitosan-based materials. In these films, the amine groups in chitosan interacted with acidic components present in the chitosan solution, which impacted the pH of the final product [47]. Increasing the concentration of chitosan raises pH due to its basic nature. Chitosan molecules can become protonated, which reduces the availability of free hydrogen ions within the matrix and, as a result, slightly elevates pH. In contrast, ascorbic acid is a weak acid that tends to decrease the pH of the matrix as its content increases. Although the linear effect of glycerol is also significant, its effect is less prominent than that of ascorbic acid and chitosan. Glycerol, a polyol, can indirectly influence the film’s pH through interactions with other acidic or basic film components [48]. It has been suggested that introducing glycerol to the system enables it to form hydrogen bonds with chitosan, displacing acetic acid in the casting solution [48]. The free acetic acid then evaporated during the drying process. However, in this study, ascorbic acid, replaced by glycerol, remained within the matrix. Therefore, increasing the glycerol content could potentially lead to a slight decrease in the surface pH of the obtained films.

#### 3.1.4. Optimized Composition for Film Preparation

**Figure 6:**
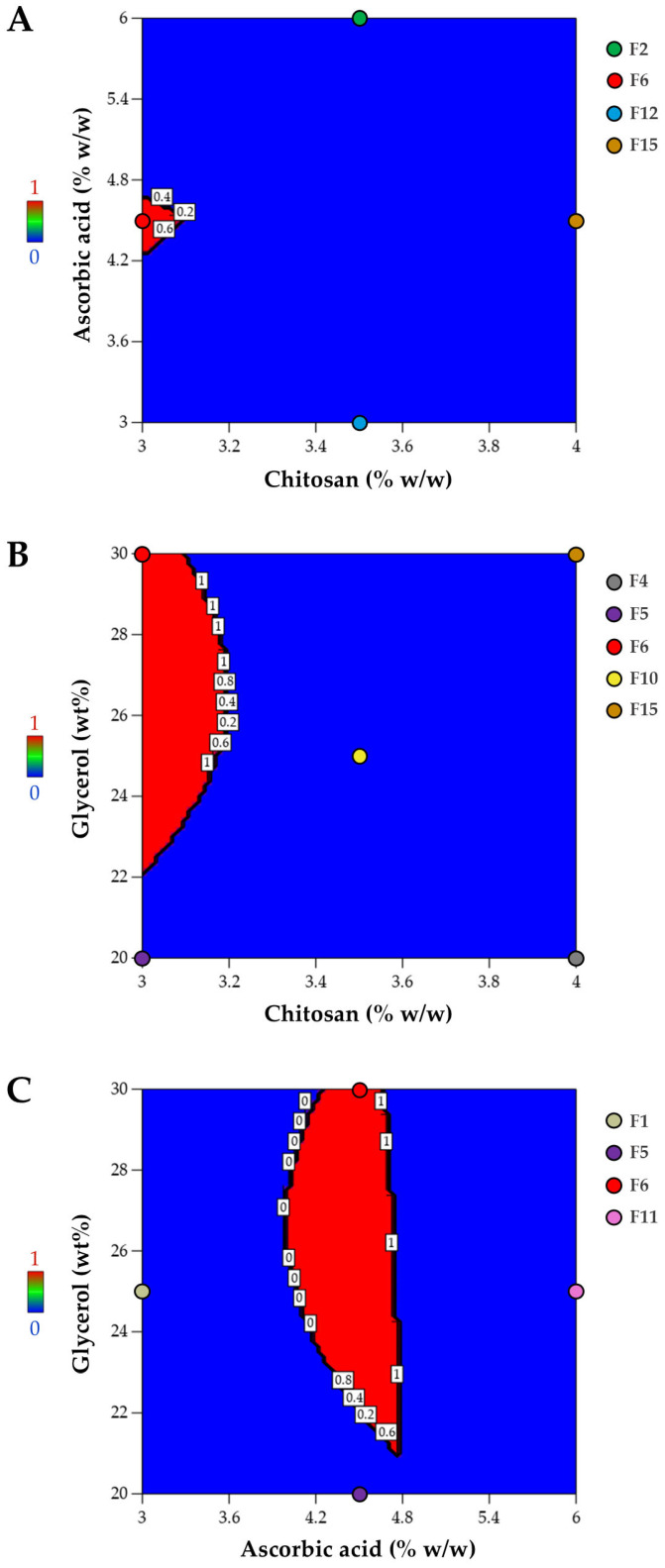
Desirability plot for optimization as a function of (**A**) chitosan and ascorbic acid, (**B**) chitosan and glycerol, and (**C**) ascorbic acid and glycerol.

Mechanical properties are a critical aspect to consider in film development, as the potential wound care material must ensure effective protection of the skin wound from external pressure while maintaining flexibility. The mechanical characteristics of the preferred film matrix should match the physical strength and flexibility of the skin. The mechanical properties of human skin vary based on factors such as age, gender, and body region. Typically, the tensile strength of skin ranges from 2.5 to 30 MPa, while its elongation capacity falls between 10% and 115% [49,50]. The ultimate strain of normal skin has been reported to have an average value of approximately 100% [49]. The acceptable range of ultimate tensile strength for materials suitable for wound care applications has been reported to be approximately 4 to 30 MPa [51]. Besides the mechanical attributes, the surface pH of topical films should also be considered. Wounds with a neutral to alkaline pH promote microbial growth; therefore, an acidic pH is crucial for enhancing wound healing [52,53]. Films with slightly low pH characteristics may serve as an acidic matrix, potentially minimizing bacterial invasion and promoting fibroblast proliferation during wound healing [54]. In addition, the pH of the film surface should be between 4.1 and 5.8, which is suitable for topical application to avoid dermal irritations [55]. Following the ANOVA analysis, numerical optimization was performed to determine the optimized composition of the casting solution formulation for film preparation, ensuring that the responses remained within the specified desired ranges: ultimate tensile strength between 4 and 30 MPa, elongation at break from 90 to 115%, and pH levels ranging from 4.1 to 5.8. The optimized formulation was identified using a desirability function, which assigns values from 0 (least favorable) to 1 (most favorable) [56]. In this approach, numerical optimization locates the point that maximizes the desirability value. As illustrated in the desirability plot (Figure 6), desirability spans from zero outside the acceptable limits to one at the optimal goal (shown in the red area). Figure 6 presents desirability plots illustrating the region where the optimized composition is located in the red area. The optimized composition was found to be F6, of which the coded levels for X_1_, X_2_, and X_3_ are −1, 0, and +1, respectively. So, the chosen formulation for further characterization was made up of 3.0% *w*/*w* chitosan, 4.5% *w*/*w* ascorbic acid, and 30.0 wt% glycerol relative to chitosan content. In addition to the film properties considered in film development, information about the casting solution’s viscosity, which may be relevant to film formation, can be beneficial for film preparation. Reported viscosities of solutions in small-scale film production range widely, from 1.7 to 1843 mPa·s [57]. In this study, preparation of the film-casting solution using the optimized composition resulted in an average viscosity of 587.3 ± 12.7 mPa·s, which falls within the previously reported suitable viscosity range of polymer solutions for film casting, approximately 50–3000 cP (0.05–3 Pa·s) [58].

### 3.2. Characterization of Film with Optimized Composition

#### 3.2.1. FTIR Analysis

**Figure 7:**
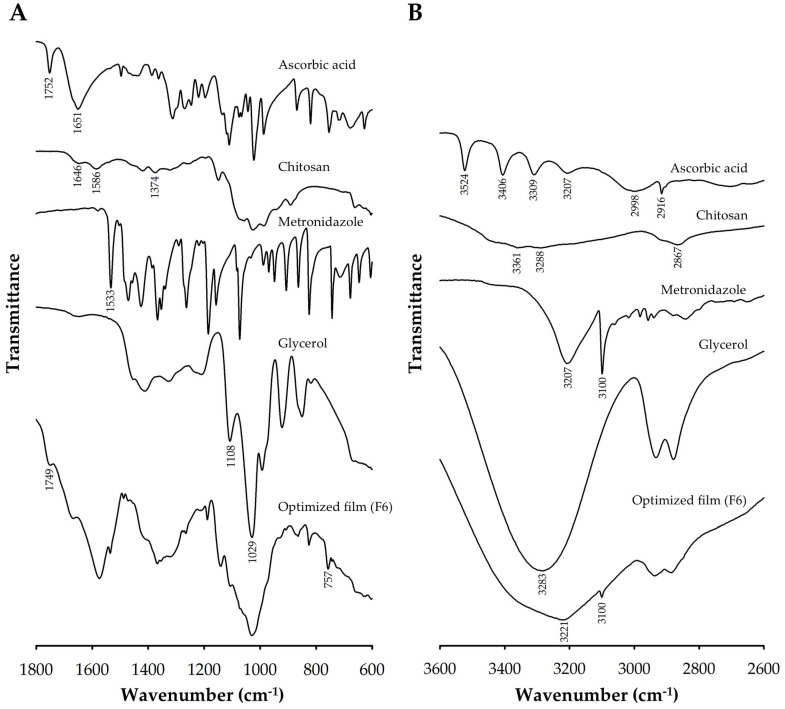
Infrared spectra of each pure compound and the optimized film over the region of (**A**) 1800–600 cm^−1^ and (**B**) 3600–2600 cm^−1^.

The FTIR spectra of each pure component and the film with optimized composition over the regions of 1800–600 cm^−1^ and 3600–2600 cm^−1^ are represented in Figure 7A and Figure 7B, respectively. According to Figure 7A, the FTIR spectrum of ascorbic acid gives the characteristic peak at 1752 cm^−1^ attributed to stretching of C=O of the five-membered lactone ring and the peak at 1651 cm^−1^ due to C=C stretching [59,60]. As shown in Figure 7B, the spectrum of ascorbic acid shows four peaks between 3560 and 3160 cm^−1^. These peaks correspond to the different hydroxyl groups. There is also a broadband centered at 2998 cm^−3^ and a peak at 2916 cm^−1^ that comes from C-H stretching vibrations. The FTIR spectrum of chitosan in Figure 7A shows the characteristic peaks at 1646, 1586, and 1374 cm^−1^ that are attributed to amide I, amide II, and amide III, respectively [61]. The broadband found in the spectrum of chitosan at the wavenumber range above 3000 cm^−1^ corresponds to the overlap of O-H and N-H stretching vibrations [61,62]; the peak at 2867 cm^−1^ is associated with C-H stretching, as can be seen in Figure 7B [61]. For the spectrum of metronidazole (Figure 7A), the characteristic peak found at 1533 cm^−1^ corresponds to N=O stretching. Over the region of 3600–2600 cm^−1^ (Figure 7B), the characteristic peaks of metronidazole are observed at 3207 cm^−1^ assigned to OH stretching, and at 3100 cm^−1^ attributed to C=C-H stretching of the imidazole ring [63,64]. For glycerol, the spectral range above 2600 cm^−1^ (Figure 7B) shows a doublet peak characteristic in the wavenumber range of 2800–3000 cm^−1^ assigned to the symmetric and asymmetric C-H stretching vibrations and a broadband centered around 3283 cm^−1^ corresponding to O-H stretching. The peaks at 1029 and 1108 cm^−1^ found in the spectrum of glycerol in Figure 7A are associated with the C-O stretching vibrations of primary alcohol and secondary alcohol in glycerol, respectively [65].

The FTIR spectrum of the optimized film is also represented in Figure 7. The broad peak at approximately 3221 cm^−1^, as labeled in Figure 7B, is due to the overlapping of the OH and NH_2_ groups of chitosan in the film [18]. The peak position of this broadband in the spectrum of the film is lower than that of plain chitosan, indicating the reduction of the free NH_2_ of the chitosan molecule following the complexation and the formation of chitosan ascorbate [18,66]. The doublet characteristic peak of glycerol can be observed in the wavenumber range of 2800–3000 cm^−1^ of the spectrum of the film. Although some of metronidazole’s distinct spectral features were not observable due to band overlap, the observed peak at 3100 cm^−1^ corresponds to metronidazole’s characteristic peak, revealing the presence of dispersed metronidazole in the film. According to Figure 7A, the characteristic peak of ascorbic acid at 1752 cm^−1^ is absent in the spectrum of the prepared film, but a peak with reduced intensity was found at the lower wavenumber around 1749 cm^−1^. The shift of the stretching peak to a lower wavenumber centered around 1749 cm^−1^ may be attributed to the complexation of ascorbic acid with chitosan and the conversion of ascorbic acid to chitosan ascorbate [17,67,68]. Moreover, the weak peak at 757 cm^−1^ reflected the C=C bending vibration of the ascorbate [17,69]. In addition, the C-O stretching vibrations associated with primary and secondary alcohols in pure glycerol merged into a single peak in the film, indicating hydrogen bonding interactions between the hydroxyl groups of chitosan and glycerol [62].

#### 3.2.2. DSC Analysis

**Figure 8:**
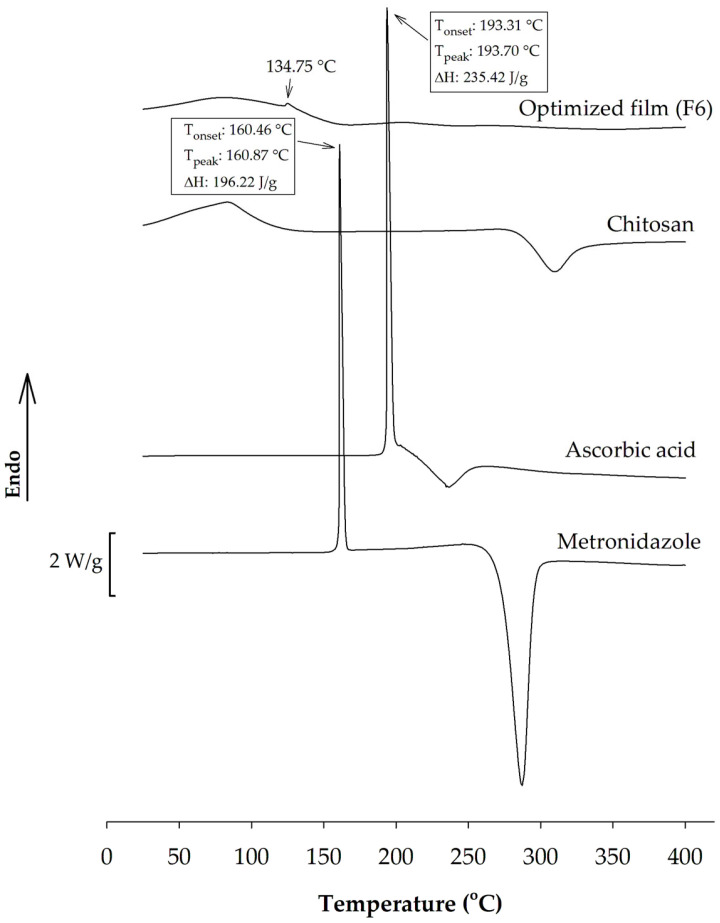
DSC thermograms of each pure solid component and the optimized film. Melting onset temperature (T_onset_), melting peak temperature (T_peak_), and melting enthalpy (ΔH) of each pure component are also shown.

The DSC thermograms of each pure solid component and the optimized film are shown in Figure 8. The DSC curve of chitosan exhibited two thermal events: the loss of bound water, accompanied by an endothermic transition at approximately 84 °C, and the decomposition, accompanied by an exothermic transition at a high temperature of approximately 310 °C. These two observed thermal transitions have also been reported previously [70]. According to Figure 8, the DSC thermogram of ascorbic acid showed a sharp endothermic melting peak at 193.70 °C, followed by thermal decomposition with the exothermic peak around 237 °C [71,72]. The DSC curve of metronidazole represented the endothermic peak at 160.87 °C and the exothermic peak around 287 °C. This endothermic event corresponded to the melting of metronidazole, while the exothermic peak above 250 °C reflected the thermal decomposition [73].

The DSC curve of the optimized film is also represented in Figure 8. Most of the DSC traces found in the individual component were not found in the film’s DSC thermogram. The absence of the melting peak of ascorbic acid in the film may be ascribed to the dispersion of amorphous ascorbic acid within the matrix and the establishment of a complex between chitosan and this bioactive compound via ionic interactions [74,75]. A small endothermic peak centered around 134.75 °C was noted, indicating that the melting point of metronidazole in the film has shifted to lower temperatures than pure metronidazole. The decrease in the melting point of metronidazole within the polymeric network may be attributed to a reduction in the crystallinity of metronidazole due to its encapsulation within the carrier matrix [76].

#### 3.2.3. PXRD Analysis

**Figure 9:**
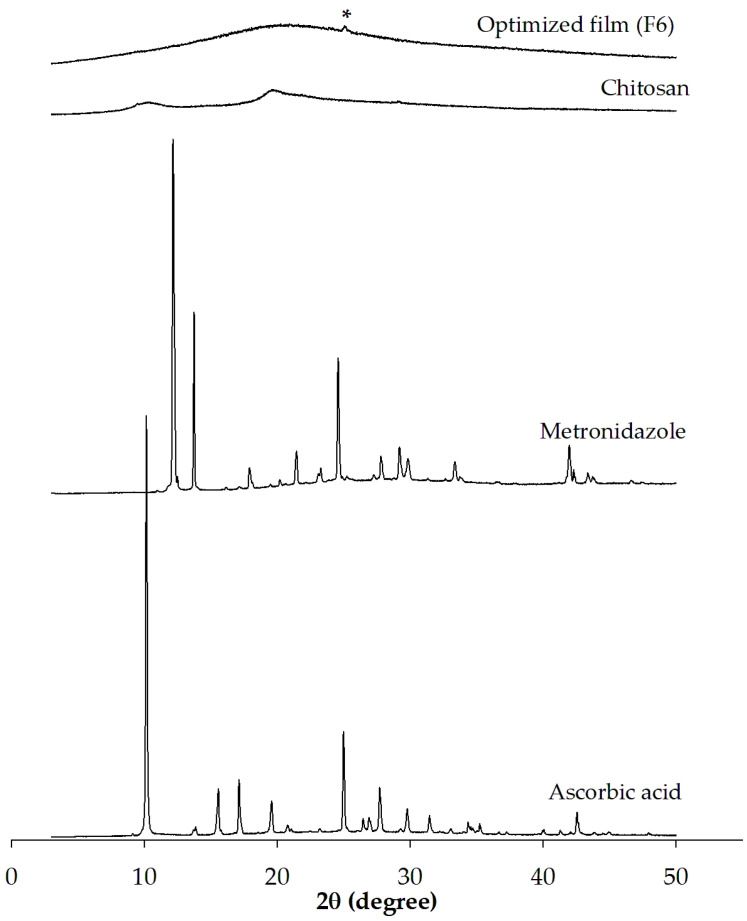
PXRD patterns of each pure solid component and the optimized film. An asterisk (*) indicates a low-intensity diffraction peak observed in the optimized film.

The PXRD patterns of each pure solid component and the optimized film are shown in Figure 9. The chitosan powder exhibited a semicrystalline state, and the diffractogram of chitosan presented an almost halo pattern [77]. The PXRD pattern of ascorbic acid demonstrated that it is a crystalline substance, as evidenced by multiple sharp diffraction peaks that correspond to the crystalline diffraction lines described in the literature [78]. According to the diffractogram of metronidazole, the multiple sharp reflections reflected the crystalline form of the drug. Although a broad and low-intensity diffraction peak at 25.05 2ϴ, as indicated by the asterisk in the figure, can be observed in the diffractogram of the film sample, most of the sharp peak characteristics of both active compounds disappeared. The absence of characteristic peaks of ascorbic acid in the polymer matrix has been reported. It can be attributed to interactions between the polymers and ascorbic acid, potentially impeding the formation of the crystalline structure of the dispersed small molecules within the polymer matrix [79]. The observed low-intensity diffraction peaks indicate the presence of the drug with a low crystallinity within the film [79]. This observation agrees with the DSC results in the preceding subsection, which revealed the reduction in the drug crystallinity in the film formulation.

#### 3.2.4. Swelling Capacity

**Figure 10:**
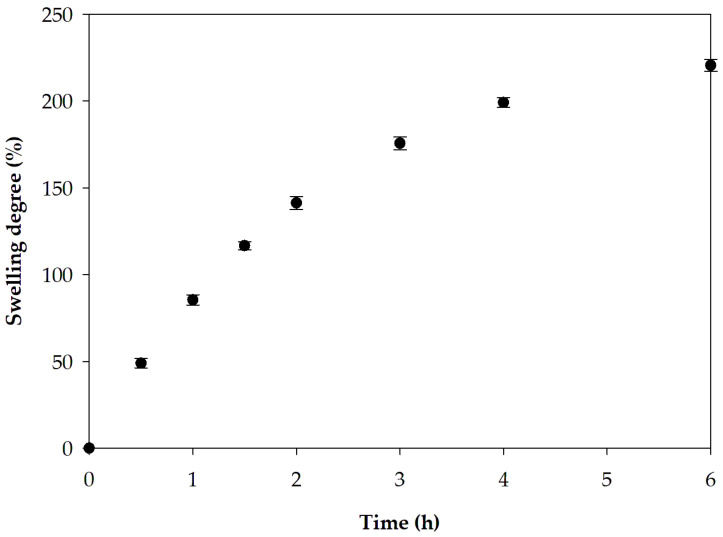
Swelling profile of the optimized film.

The swelling ability of the topical films enables the film to absorb wound exudates [32,33]. The swelling capacity of the optimized film at different times is displayed in Figure 10. The values of the swelling degree of the prepared samples increased over time. At 6 h, the swelling degree reached 220.6%. The swelling degree increases rapidly in the early stages of the test and becomes nearly comparable at later time points. The swelling capacity of the prepared film is potentially attributed to the presence of polar groups within the polymer network that can interact with water [32,80]. This fluid absorption capability is favorable for wound care applications [32,33].

#### 3.2.5. Drug Content

The mean drug content percentages, reflecting the encapsulation efficiency of the active compounds in the prepared film [24,34], were 91.9% and 98.4% for ascorbic acid and metronidazole, respectively. The obtained values suggest that the active compounds were retained effectively, with minimal loss during the film preparation process [24].

#### 3.2.6. In Vitro Release

**Figure 11:**
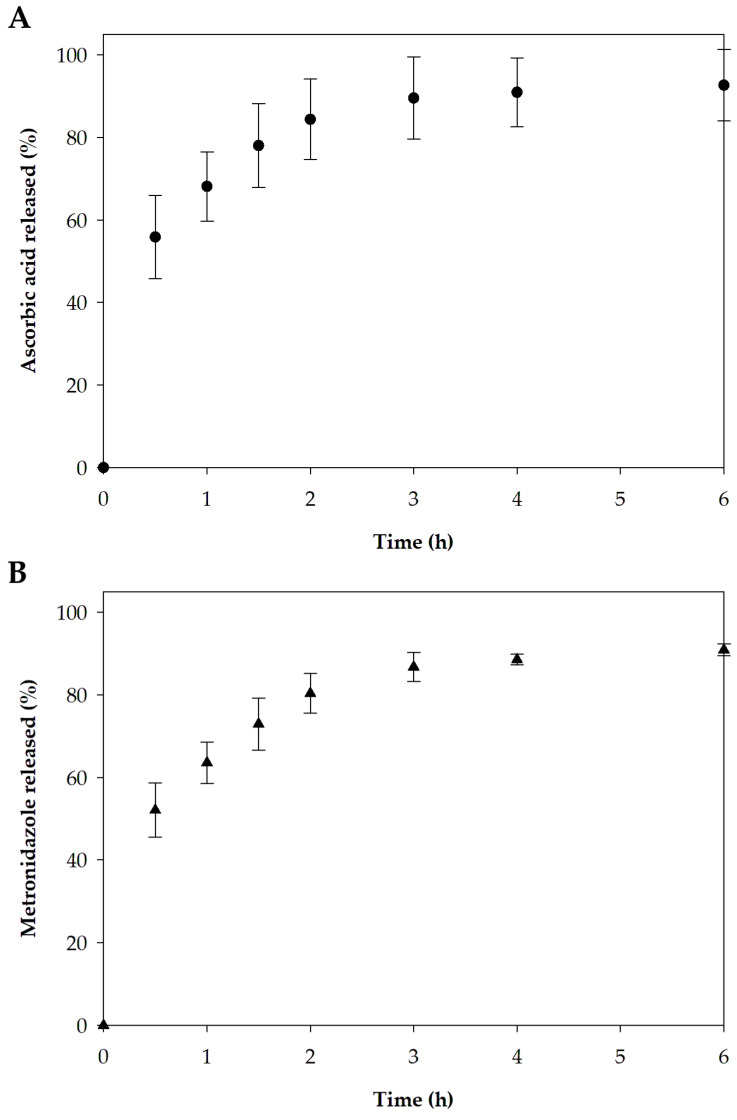
The release profiles of (**A**) ascorbic acid and (**B**) metronidazole from the optimized film.

The release of active pharmaceutical ingredients from a pharmaceutical drug product to the target site is critical for achieving the desired therapeutic benefits [81]. The release profiles of ascorbic acid and metronidazole from the optimized film formulation are presented in Figure 11. An initial rapid release followed by the sustained release of the remaining active molecules in the film was observed for both active compounds. The release of ascorbic acid and metronidazole in 6 h reached 92.7% and 90.9%, respectively. The immediate release of the drug in the early period may be associated with drug diffusion resulting from the rapid swelling of the polymeric film [81]. In addition to the film’s swelling characteristic, the low-crystallinity or amorphous form of both pharmaceutical compounds also contributes to their readiness for release upon application. After rapid release, there is sustained release because the swollen matrix slows the movement of the compounds trapped in the film [81,82]. For wound application, an initial burst release is desirable to ensure drug delivery to the lesion promptly after the dosage form administration [82]. After the rapid release, a sustained release of the active compounds helps maintain therapeutic efficacy [82,83].

Considering the two active compounds studied in this research, ascorbic acid and metronidazole, the observed co-release and the obtained release pattern, characterized by an initial burst followed by a sustained release phase, are advantageous for topical applications. For ascorbic acid, Khaloo Kermani et al. proposed that delivering the compound in a burst mode at the beginning of treatment may be beneficial for promoting wound contraction, wound closure, and angiogenesis [84]. Furthermore, the gradual release of ascorbic acid for up to 6 h after the initial burst is also desirable in dermatological applications, as it facilitates the maintenance of therapeutic benefit over time [85]. For metronidazole, the initial burst release from topical carriers may enhance therapeutic efficacy by enabling rapid delivery of the drug to the site of application shortly after administration [86]. A biphasic release profile, consisting of an initial burst followed by sustained release of metronidazole, has demonstrated promising potential for sustained delivery applications, as previously reported by Shinde et al. [87]. In addition to the release characteristics, the favorable swelling capacity—indicative of exudate absorption capability—suggests the potential of the prepared formulation for topical application at wound sites [88]. As part of future work, conducting stability studies would be beneficial for supporting shelf-life determination and guiding further development of the film formulation for potential commercial application.

## 4. Conclusions

A chitosan-based film plasticized with glycerol was successfully developed for the topical delivery of ascorbic acid and metronidazole, without requiring additional mineral or organic acids in the casting solution. Both glycerol and ascorbic acid contributed to film plasticization, while FTIR analysis revealed the formation of chitosan ascorbate and interactions between chitosan and glycerol. Moreover, DSC and PXRD data demonstrated alterations in the thermal characteristics and crystallinity of the embedded bioactive compounds. The optimized film exhibited favorable swelling capacity and simultaneously released ascorbic acid and metronidazole. These findings suggest that this chitosan-based film could be a promising platform for topical wound delivery applications.

## Figures and Tables

**Table 1:** Factors and their levels in Box–Behnken design–response surface methodology.

Factors	Levels		
	Low (−1)	Medium (0)	High (+1)
Chitosan (X_1_)	3	3.5	4
Ascorbic acid (X_2_)	3	4.5	6
Glycerol (X_3_)	20	25	30

**Table 2:** Box–Behnken design matrix.

Formulations	Coded Levels of Independent Factors		
	X_1_	X_2_	X_3_
F1	−1	−1	0
F2	0	+1	+1
F3	0	0	0
F4	+1	0	−1
F5	−1	0	−1
F6	−1	0	+1
F7	0	0	0
F8	0	0	0
F9	0	+1	−1
F10	0	0	0
F11	−1	+1	0
F12	0	−1	+1
F13	0	−1	−1
F14	0	0	0
F15	+1	0	+1
F16	+1	+1	0
F17	+1	−1	0

**Table 3:** Fit summary of regression analysis results for the ultimate tensile strength (Y_1_), elongation at break (Y_2_), and pH (Y_3_).

Responses	Models	Sequential *p*-Value	R^2^	Adjusted R^2^	Predicted R^2^	Lack-of-Fit *p*-Value	Remarks
Ultimate tensile strength (Y_1_)	Linear	0.0011	0.6997	0.6303	0.3831	0.0086	
	**2FI**	**0.0009**	**0.9377**	**0.9003**	**0.7292**	**0.0864**	**Suggested**
	Quadratic	0.7616	0.9467	0.8782	0.2656	0.0419	
Elongation at break (Y_2_)	Linear	<0.0001	0.8598	0.8275	0.7258	0.1135	
	2FI	0.6260	0.8814	0.8102	0.4703	0.0834	
	**Quadratic**	**0.0131**	**0.9722**	**0.9365**	**0.7762**	**0.4495**	**Suggested**
pH (Y_3_)	Linear	0.0069	0.5945	0.5009	0.2846	0.1155	
	2FI	0.1135	0.7707	0.6331	0.3765	0.1730	
	**Quadratic**	**0.0151**	**0.9440**	**0.8719**	**0.7485**	**0.7997**	**Suggested**

**Table 4:** Estimated coefficients of coded factors and ANOVA analysis for the model suggested by Box-Behnken design-response surface methodology.

Source	Ultimate Tensile Strength (Y_1_)		Elongation at Break (Y_2_)		pH (Y_3_)	
	Coefficient	*p*-Value	Coefficient	*p*-Value	Coefficient	*p*-Value
Model		<0.0001		0.0001		0.0013
Intercept	4.34	-	69.85	-	4.31	-
X_1_	−0.5228	0.1487	−24.13	0.0001	0.0450	0.0036
X_2_	−3.12	<0.0001	39.55	<0.0001	−0.0725	0.0002
X_3_	−1.59	0.0008	9.63	0.0200	−0.0300	0.0244
X_1_X_2_	1.91	0.0023	−9.91	0.0658	0.0550	0.0076
X_1_X_3_	−1.08	0.0447	−3.66	0.4468	0.0150	0.3459
X_2_X_3_	1.92	0.0023	0.8643	0.8546	−0.0400	0.0309
X_1_^2^	-	-	10.43	0.0508	0.0405	0.0266
X_2_^2^	-	-	−0.7586	0.8689	0.0505	0.0101
X_3_^2^	-	-	−18.94	0.0037	0.0055	0.7151

## Data Availability

The original contributions presented in this study are included in the article; further inquiries can be directed to the corresponding author.
